# Localization-assisted stimulated Brillouin scattering spectroscopy

**DOI:** 10.1063/5.0087697

**Published:** 2022-05-03

**Authors:** Giulia Zanini, Giuliano Scarcelli

**Affiliations:** Fischell Department of Bioengineering, University of Maryland, 8278 Paint Branch Dr., College Park, Maryland 20742, USA

## Abstract

Brillouin spectroscopy has emerged as a promising modality to noninvasively probe the mechanical properties of biologically relevant materials. Stimulated Brillouin scattering (SBS) has the potential to improve measurement speed and resolution by exploiting a resonant amplification of the scattered signal, yet current SBS spectrometers have not provided significant improvements due to fundamental and practical limitations of illumination and detection parameters. To overcome this challenge, here we derive a signal localization theory for the Brillouin spectral domain and accordingly design an SBS spectrometer with much improved performances compared to state-of-the-art systems. We present experimental and simulated data validating our theory, which result in a tenfold improvement in acquisition speed, or an order of magnitude improved spectral precision, for SBS spectral measurements when properly optimizing the SBS photon detection architecture.

## I. INTRODUCTION

Over the past ten years, Brillouin spectroscopy has emerged as a promising modality to noninvasively probe the mechanical properties of biological samples.^1^ Thanks to its all-optical, contact-free, and label-free nature, Brillouin spectroscopy allows for *in vivo* 3D mapping of longitudinal elastic modulus distribution within cells and tissues, even in settings where physical contact is not possible, such as in 3D matrices or microfluidics channels, providing sufficient sensitivity to detect and monitor relevant biomechanical changes.^2–7^

Current Brillouin technology relies on the spontaneous Brillouin scattering interaction in which photons are inelastically scattered by the acoustic phonons intrinsically propagating in the material, thus acquiring a characteristic frequency shift and spectral broadening that can be linked to the local complex longitudinal modulus.^8^ Due to the nature of the interaction, the Brillouin signal is technically challenging to detect. It is typically only few GHz (<0.01 nm) away from the incident laser light, it exhibits a linewidth on the order of hundreds of MHz, and it is many orders of magnitude weaker than elastic scattering or back-reflections. For this reason, despite the tremendous progress in spectrometers featuring high throughput, spectral extinction, and resolution,^9^ Brillouin spectrum acquisition is still limited to 20–100 ms in biological samples.^10,11^ A great deal of attention is currently dedicated to overcoming this limitation in Brillouin spectroscopy. A line-scanning architecture was recently demonstrated,^12^ in which the use of a line-beam illumination and a 90°-geometry detection allows for the simultaneous spectral analysis of hundreds of points but with limited spectral extinction and resolution.

An intriguing alternative approach relies on exploiting the stimulated version of Brillouin scattering, a non-linear process in which the coupling of optical and acoustic fields could potentially yield five orders of magnitude stronger signal and intrinsically free from the elastic background.^13^ In stimulated Brillouin scattering (SBS), a resonant amplification of the acoustic wave is achieved through the interaction of two counterpropagating laser beams—pump and probe—whose frequency difference matches the phonon frequency. As a result, the inelastic scattered signal experiences an exponential increase, which can be detected as a gain in the probe transmission. The Brillouin spectrum can then be traced by scanning the probe frequency around the resonance without spectral dispersive elements, thus allowing access to linewidth measurements with high spectral resolution limited only by the laser linewidth. To sensitively detect the probe gain, a lock-in detection is customarily used and can lead to shot-noise limited operation.^14^ With this scheme, biomechanical imaging of small organisms was recently demonstrated^15^ in which both elastic and viscous components of the longitudinal modulus were accessed through the spectral analysis of the SBS spectrum with signal-to-noise ratio (SNR) and precision comparable to spontaneous Brillouin. However, modest speed improvements were demonstrated with the exposure time of 20 ms required to retrieve the SBS spectrum. These performances are fundamentally limited by the CW operation, which leads to weak non-linear interactions, but pulsed operation is not straightforward to implement for SBS in biological samples because of the simultaneous requirements of narrow linewidth, repetition rate, and energy dose as well as the availability of suitable laser sources.

To advance SBS spectroscopy, here we derive a signal localization theory for the Brillouin spectral domain, and accordingly design an SBS system with much improved performances compared to state-of-the-art SBS spectrometers. We present experimental and simulated data validating our theory, which results in a tenfold improvement in acquisition speed and an order of magnitude improved spectral precision for SBS spectral measurements.

## II. PRINCIPLE AND THEORY

### A. Brillouin scattering

Brillouin scattering is a type of inelastic scattering, which involves the interaction of photons with thermally generated and propagating density fluctuations in a material, also referred to as acoustic phonons.^16^ As a result, the scattered photons acquire a Lorentzian-distributed frequency shift with central shift ν_B_ and linewidth Γ_B_ given by

$$\begin{array}{c} \nu_{\text{B}}=2n\frac{v_{s}}{c}\nu \sin\frac{\theta }{2}\\ 2\pi \Gamma_{\text{B}}=4\pi^{2}{\left(\frac{2n\nu }{c}\sin\frac{\theta }{2}\right)}^{2}\Gamma^{\prime } \end{array},$$ (1)

where n is the material refractive index, v_s_ is the speed of sound, c is the speed of light, ν is the incident photon frequency, θ is the scattering angle, and Γ′ is the damping parameter.

The spectral features of the scattered light reflect the viscoelastic properties of the material, and in the case of backscattering geometry, the real and imaginary parts of the complex longitudinal modulus, M′ and M″, respectively, can be written as^13^

$$\begin{array}{c} M^{\prime }=\rho v_{s}^{2}=\frac{\rho }{n^{2}}\frac{\nu_{\text{B}}^{2}c^{2}}{4\nu^{2}},\\ M^{\prime \prime }=\rho v_{s}^{2}\frac{\Gamma_{\text{B}}}{\nu_{\text{B}}}=\frac{\rho }{n^{2}}\frac{\nu_{\text{B}}\Gamma_{\text{B}}c^{2}}{4\nu^{2}}, \end{array}$$ (2)

where ρ is the mass density. Interestingly, an empirical relation between the Brillouin-extracted storage modulus M′ and the typically measured Young’s modulus E′ has been demonstrated in several biological systems.^2,17,18^

In SBS, the amplification of the acoustic wave, and, thus, of the scattering interaction, is achieved when the frequency difference Δν = |ν_1_ − ν_2_| between pump (ν_1_) and probe (ν_2_) beams matches the phonon frequency ν_B_. The SBS signal is contained in the gain of the transmitted probe beam, which, for weak non-linearities, can be approximated as^13^

$$G\left(\Delta \nu \right)=\frac{\Delta I_{2}\left(\Delta \nu \right)}{I_{2}}=g\left(\Delta \nu \right)I_{1}L,$$ (3)

where ΔI_2_ is the intensity variation of the probe beam due to the SBS interaction, I_1_ and I_2_ are the injected pump and probe intensities, respectively, L is the interaction length, and

$$g\left(\Delta \nu \right)=g_{0}\frac{{\left(\Gamma_{\text{B}}/2\right)}^{2}}{{\left(\Delta \nu -\nu_{\text{B}}\right)}^{2}+{\left(\Gamma_{\text{B}}/2\right)}^{2}}$$ (4)

is the Lorentzian gain factor centered at ν_B_ with full width at half maximum Γ_B_ and amplitude,

$$g_{0}=\frac{\left(2\pi \right)\gamma_{e}^{2}\nu^{2}}{nv_{s}c^{3}\rho \Gamma_{B}}=\frac{\left(4\pi \right)\gamma_{\text{e}}^{2}\nu^{3}}{c^{4}\rho \nu_{\text{B}}\Gamma_{\text{B}}},$$ (5)

which depends on the incident optical frequency ν = ν_1_ ∼ ν_2_ and on electrostrictive constant γ_e_, refractive index n, and mass density ρ proper of the sample.

### B. Localization theory

As introduced in Sec. II A, Brillouin spectroscopy can be used to assess the longitudinal storage and loss moduli from the frequency shift ν_B_ and the linewidth Γ_B_ of the scattered light. This is typically achieved through least-square fitting of the data acquired with a spectrometer, aiming at the estimation of the spectral features committing the smallest error possible.^19^ This type of analysis of the Brillouin spectrum can be thought of as a localization problem in the spectral domain.

Localization problems have recently attracted much attention mainly for their role in the development of super-resolution microscopy techniques such as photoactivated localization microscopy (PALM) and stochastic optical reconstruction microscopy (STORM), in which the position of fluorescent probes is estimated applying fitting algorithms to 2D diffraction-limited images.^20^ At the basis of localization microscopy is the fact that the position of the center of sparse, sub-resolved objects can be estimated with arbitrary precision given a sufficient number of photons, even if their size is much smaller than the microscope point spread function (PSF).^21^ Localization precision scales as the noise level present in the signal, which is strongly dependent on the number of signal photons N. The two main sources of noise are shot noise coming from the signal, which scales as 1/√N; and background noise coming from sources other than the signal, which scales as 1/N. At the high photon budget, shot noise is dominant, while at low light levels, background noise prevails. Because of the use of a camera system in detection, a third noise contribution must be added, which is the pixelation noise arising from the uncertainty on where each individual photon arrives in the camera pixel. This noise scales as the pixel size *a* and can be added in quadrature to the other noise sources. Intuitively, the larger the pixel size, the higher the number of collected photons per pixel, which can help overcoming the background noise and improving precision. However, when the pixel size becomes comparable or larger than the PSF, the information on the spatial distribution of the photons is progressively lost and, as a result, the uncertainty on the center position increases.

Here, we derive the localization theory for the spectral domain of SBS detection systems, focusing, in particular, on the estimation of the peak position ν_B_ from SBS spectra, the most used Brillouin signature in current biomechanical applications for the extraction of the storage modulus.

**FIG. 1.**
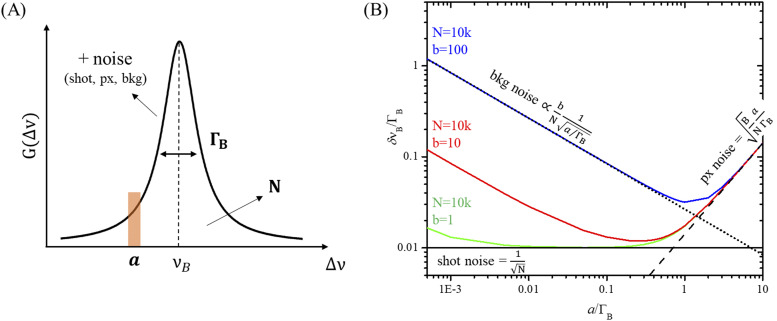
(a) Representation of an SBS gain spectrum G acquired scanning the laser frequency difference Δν, exhibiting a Lorentzian shape centered at ν_B_ with broadening Γ_B_. The experimental parameters, which affect peak localization are highlighted: the number of Brillouin photons N, linewidth Γ_B_, channel size *a*, and noise. (b) Simulated behavior of the normalized peak localization precision δν_B_/Γ_B_ as a function of the normalized channel size *a*/Γ_B_ computed using Eq. (7) with N = 10k, B = 2 and with different background noise levels b. The single noise components are plotted for reference for case b = 100 (blue line): shot noise (solid black line), pixelation noise (dashed black line) and background noise (dotted black line).

In SBS, the spectrum is obtained by scanning the probe frequency around the resonance, while the gain signal, extracted with the lock-in amplifier (LIA) from the total transmitted probe intensity, is sampled to reconstruct the characteristic Lorentzian peak shape. The total number of SBS photons N is reflected in the area under the peak, the shot noise scales as 1/√N, and the background noise originates from pump stray light. In this configuration, the equivalent of the pixel size *a* of a camera-based system is the spectral channel size defined as the frequency range analyzed by the lock-in amplifier to extract a single SBS signal sample. The larger the channel, the more photons collected per channel, but the higher the uncertainty on the exact frequency of the individual photons. This “pixelation” noise becomes dominant at channel sizes that approach and exceed the spectrometer PSF. In SBS, being the spectral resolution limited only by the laser linewidth, which is much smaller than the typical Brillouin linewidth, the spectral PSF is given by the natural broadening Γ_B_, which is sample-dependent [Fig. 1(a)].

In this framework, the error in peak localization δν_B_ can be written as

$${\left(\delta \nu_{\text{B}}\right)}^{2}=\frac{\Gamma_{\text{B}}^{2}+Ba^{2}}{N}+\frac{4\sqrt{\pi }\Gamma_{\text{B}}^{3}b^{2}}{aN^{2}},$$ (6)

where Γ_B_ is the natural Brillouin linewidth, N is the total number of Brillouin photons, B is a broadening factor, which describes pixelation noise, *a* is the spectral channel size, and b is the number of background noise photons. The first term contains the dependence of precision on the photon-counting noise coming from shot and pixelation noise, while the second term contains the background noise contribution, which, for the sake of generality, has been derived considering a Gaussian distribution of Brillouin photons.^21^

The precision equation [Eq. (6)] can be factorized in the following form:

$$\delta \nu_{\text{B}}=\frac{\Gamma_{\text{B}}}{\sqrt{N}}\sqrt{1+B{\left(\frac{a}{\Gamma_{\text{B}}}\right)}^{2}+\frac{4\sqrt{\pi }b^{2}}{N}\frac{1}{a/\Gamma_{\text{B}}}}$$ (7)

where the dependence on the ratio channel size *a* over linewidth Γ_B_ is made explicit. Figure 1(b) illustrates this dependence under different experimental conditions, computed by varying the background noise level b while keeping a constant number of Brillouin photons N, and using a given broadening factor B. The precision (here normalized to the linewidth to remove sample dependence) clearly exhibits two distinct behaviors as the channel size *a* is varied. For *a* ≪ Γ_B_, the uncertainty in the localization monotonically decreases as the channel size increases. This regime is dominated by the background noise contribution, as seen by the plot of the second term of Eq. (6) (dotted black line, relative to blue condition): in a small channel, few signal photons are collected and the background noise prevails, degrading SNR and thus precision. As the background noise is reduced (from blue to green conditions), the overall precision improves and closely approaches the shot noise limit (solid black line). Instead, as the channel reaches and goes beyond the linewidth size, precision starts to degrade following a single behavior for all conditions, as the precision is dominated by the pixelation noise component (dashed black line, relative to blue condition). The optimal channel size, which corresponds to maximum SNR and precision, can be found at

$${\left(a/\Gamma_{\text{B}}\right)}_{\min}^{3}=\frac{2\sqrt{\pi }b^{2}}{BN}.$$ (8)

The theory shows how, in SBS spectroscopy, it is critical to design a proper detection system based on the various experimental conditions, such as the number of signal and background noise photons, to attain significant improvements in SNR and precision of the measurement. Particular attention should be made in maximizing the signal collection, which can be achieved by designing an acquisition system with the tunable spectral channel size that can be matched with the specific experimental settings.

## III. METHODS

### A. Experimental setup

**FIG. 2.**
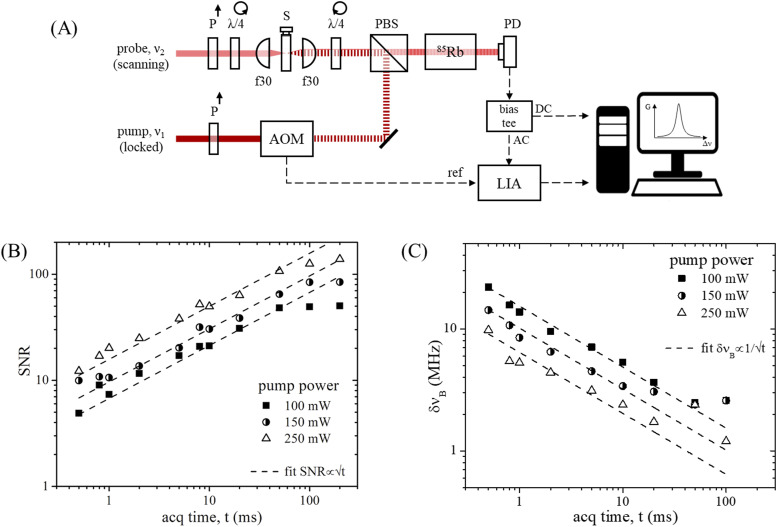
(a) Schematic representation of the SBS setup used in this study. P: polarizer, λ/4: quarter-wave plate, f: focal length (mm), S: sample, AOM: acousto-optic modulator, PBS: polarizing beam splitter, PD: photodetector, and LIA: lock-in amplifier. (b) Experimental SNR and (c) shift precision computed for 100 water spectra acquired at constant probe power (∼20 mW) with varying pump power and acquisition time. Dashed lines in (b) and (c) represent fit of the data, confirming shot noise limited performances of the system down to millisecond exposures.

Our SBS spectrometer [Fig. 2(a)] consists of two CW single-frequency tunable lasers at 780 nm (Toptica DL pro and TA pro), with ∼100 kHz linewidth and 30–50 GHz tunability range, focused by low-NA optics and overlapped in a counterpropagating geometry at the sample, which is a quartz cuvette filled with a liquid. The pump frequency is locked at a Rb85 absorption line using a vapor-based locking module available with the laser, and its intensity is modulated at 1 MHz by an acousto-optic modulator (AOMO 3080-125, Crystal Technology, Inc.). SBS spectra are obtained scanning the probe frequency around the resonance with the acoustic wave, while recording the beat frequency with the pump with a fast detector (1544-A, Newport) and a signal analyzer (N9000B, Keysight). The transmitted probe beam is detected by an amplified detector (PDA36A2, Thorlabs) from which the SBS signal is extracted using a bias tee (ZFBT-4R2GW+, MiniCircuits) and a lock-in amplifier (LIA, UHF, Zurich Instruments) referenced to the pump modulation frequency. The insertion of a hot Rb85 vapor cell (Precision Glassblowing) in detection allows for the minimization of the background photons mainly coming from pump stray light, which, being modulated, is detected by the LIA. Spectra are recorded by the data acquisition module present in the probe laser control unit, whose data sampling is synchronized with the probe frequency scanning and it can be operated at a minimum of 1000 samples/scan and at a maximum sampling rate of 200 kHz.

The system works in the weak non-linear regime for the range of powers available in our setup (Fig. S1). The SBS signal results to be proportional to the product of pump and probe powers, here expressed in the linearity with the pump power and in a constant probe power dependence of the gain, as described by Eq. (3). Moreover, the system works in the shot noise limit down to millisecond exposures, and both SNR and frequency shift precision result proportional to applied power (Fig. 2).

Overall, the performances achieved by our setup, before applying the localization-assisted design, are comparable to current state-of the-art SBS spectrometers,^15^ which display only a moderate improvement compared to spontaneous Brillouin.^10^

### B. Data acquisition

In order to experimentally validate the localization theory in the SBS spectra acquisition and analysis, the first step requires understanding how to control the main parameters, which affect shift precision—namely, the number of signals and background noise photons and spectral channel size. The number of SBS photons is proportional to applied powers and gain factor [Eq. (3)] and to the spectrum acquisition time. The background noise is primarily originating from pump stray light, which is minimized thanks to the use of a counterpropagating illumination geometry and of a hot Rb cell in detection. The spectral channel size *a*, which is defined as the frequency range from which a single SBS gain sample is extracted by the LIA, can be identified with the time constant τ_LIA_ of the digital low-pass filter, which constitutes the last stage in the LIA phase-sensitive detection method. τ_LIA_ is user-adjustable, it defines SNR and response time of the signal recovery, and it indeed covers a frequency range during the scanning-based spectrum acquisition over which it performs an exponential moving average of the input signal: the bigger the time constant τ_LIA_, the higher the SNR but the longer the signal recovery time, which means that, at fixed scan speed and spectrum acquisition time, the larger the spectral channel size *a*.

Experiments were designed to acquire and analyze SBS spectra at different channel sizes. 2-GHz water and methanol spectra were acquired at fixed pump and probe powers (250 and 20 mW, respectively), and at fixed acquisition time (5 and 100 ms, which correspond to scanning speed of 400 and 20 GHz/s, respectively, and a sampling rate of 200 and 10 kHz at 1000 samples/scan, respectively), so to keep a constant number of SBS photons under each channel condition. The spectral channel size *a* was varied over 3 orders of magnitude (from 0.1 to 400 MHz) by tuning τ_LIA_. Linewidth, peak amplitude, and peak noise were evaluated through Lorentzian fitting of 50 spectra/channel condition.

## IV. RESULTS AND DISCUSSION

### A. Experimental validation of localization theory

**FIG. 3.**
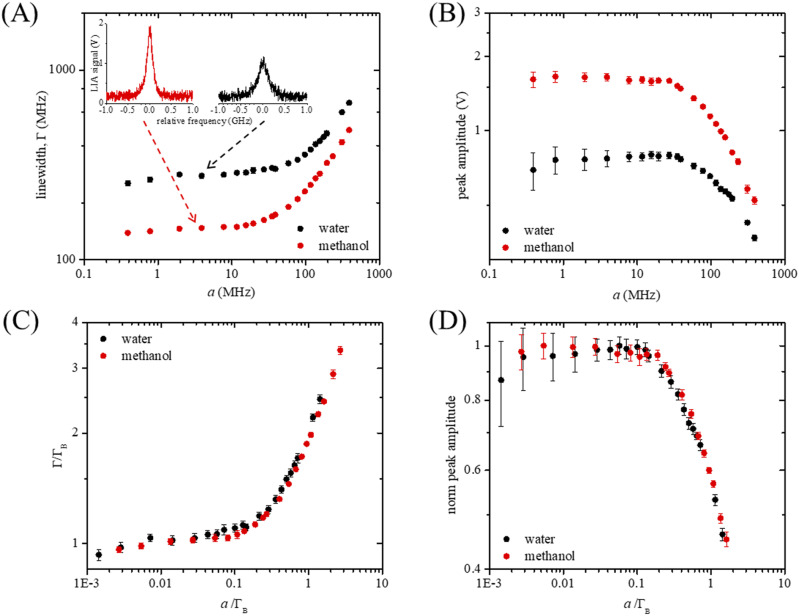
(a) and (b) Linewidth and peak amplitude of water (black) and methanol (red) SBS spectra acquired at constant acquisition time (5 ms) and constant pump and probe powers (250 and 20 mW, respectively), while varying the spectral channel size *a*. These spectral features were extracted through Lorentzian fit of 50 spectra per channel condition. Insets in (a) show two exemplary water and methanol spectra. (c) and (d) Data in (a) and (b) plotted with normalized axes in order to remove any sample dependence.

To demonstrate the universal validity of our model of SBS detection, we performed experiments with two samples, water, and methanol that have markedly different shift, linewidth, and gain. Figure 3 shows the analysis of their respective SBS spectra acquired in 5 ms at constant pump and probe powers, while varying the LIA time constant τ_LIA_ and, thus, the channel size *a*. Insets in Fig. 3(a) show a qualitative example of the acquired spectra. In Figs. 3(a) and 3(b), peak linewidth and amplitude are plotted as a function of *a*. The peaks broaden as *a* increases due to pixelation effects [Fig. 3(a)], and at the same time, their amplitude decreases [Fig. 3(b)], leading to a constant peak area (Fig. S2), which correctly reflects the experimental condition of a constant number of SBS photons. Importantly, when the x axis is scaled to the intrinsic linewidth Γ_B_ of the samples—here measured averaging the first five data points as 270 ± 10 and 144 ± 4 MHz for water and methanol, respectively—and the y axis is scaled to the specific gain of each sample (here calculated as the average of the first five data points), the dependency of the experimental results on the sample type is removed. Figures 3(c) and 3(d) show the two curves collapsing into a single behavior, which only depends on the experimental setup and on the spectral acquisition parameters.

**FIG. 4.**
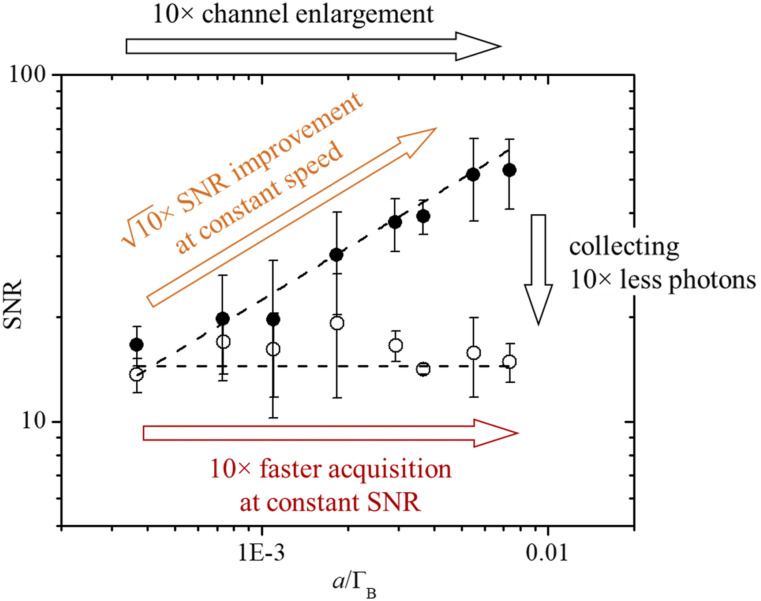
SNR evaluation of water spectra acquired at constant pump and probe powers (250 and 20 mW, respectively) while enlarging the channel size *a* over an order of magnitude. Filled circles correspond to spectra acquired at constant acquisition time (100 ms), while open circles to spectra acquired in shorter times while enlarging the channel (from 100 to 5 ms). Dashed lines fit the data with a square-root (filled circles) and a constant (open circles) behavior. This plot summarizes the double advantage of the tuning of the channel size.

Let us now demonstrate the power of designing detection parameters based on the localization theory. The noise at the peak of the spectrum, as seen in the error bars of Figs. 3(b) and 3(d), decreases as the channel size increases, which, up to the onset of pixelation, corresponds to an SNR improvement. This is explicitly shown in Fig. 4 (filled circles), where the SNR—calculated as the ratio of peak amplitude over noise at the peak—was evaluated for 50 water spectra acquired in 100 ms. The channel enlargement corresponds to an equivalent square root improvement in SNR at constant acquisition time. This is a direct consequence of the shot-noise limited regime of operation of the SBS spectrometer [Fig. 2(b)], which benefits from the collection of a larger number of photons per channel to overcome the background noise contribution.

This SNR behavior as a function of the channel size opens an intriguing measurement opportunity. If the goal of the measurement is the acquisition of spectra with a certain SNR level and precision, less photons can be collected while enlarging the channel size, or equivalently, the spectrum acquisition can be proportionally sped up, as here demonstrated in Fig. 4 (open circles) where, using a channel about ten times larger, spectra were acquired more than ten times faster, while retaining the SNR even if collecting ten times less photons.

These results prove that the performances of the SBS spectrometer can be tuned by varying the detection parameters, as predicted by the localization theory: on one side, the SNR can be improved only by increasing the channel and allowing more photons to be collected per channel, without having to increase the total acquisition time and the total number of photons; on the other hand, the acquisition time can be decreased proportionally to the channel enlargement without degrading the SNR.

### B. Brillouin shift precision

The experimental results presented in Sec. IV A are subsequently used to evaluate the shift precision δν_B_, as in the error committed to estimate the frequency shift of the Brillouin peak from the acquired spectra, a key parameter in Brillouin experiments to access mechanical properties, as underlined in Sec. II.

The values for linewidth, peak amplitude, and peak noise retrieved from the experiments [Figs. 3(a) and 3(b)] were used in the simulation of 100 Lorentzian spectra for each channel condition, centered at the nominal frequency shift for water and methanol taken from the literature, i.e., 5.1 and 3.6 GHz, respectively. The peak position was extracted through Lorentzian least-square fitting of the simulated spectra, and its standard deviation was computed for each channel size.

The choice of using simulated spectra for precision estimation was dictated by the limited sampling capabilities of the current setup (see Sec. III A). The sampling interval on a 2-GHz scan was limited to a 2-MHz maximum width, and it could not be correctly matched to the channel size determined by the LIA time constant, leading to artifacts in the signal acquisition, such as the progressive shift of the peak in the scanning direction and an asymmetrical broadening as the time constant becomes much larger than the sampling interval. Experimental data presented in Fig. 4 were indeed collected at channel sizes below 2 MHz, where also the sampling interval could be adjusted accordingly.

**FIG. 5.**
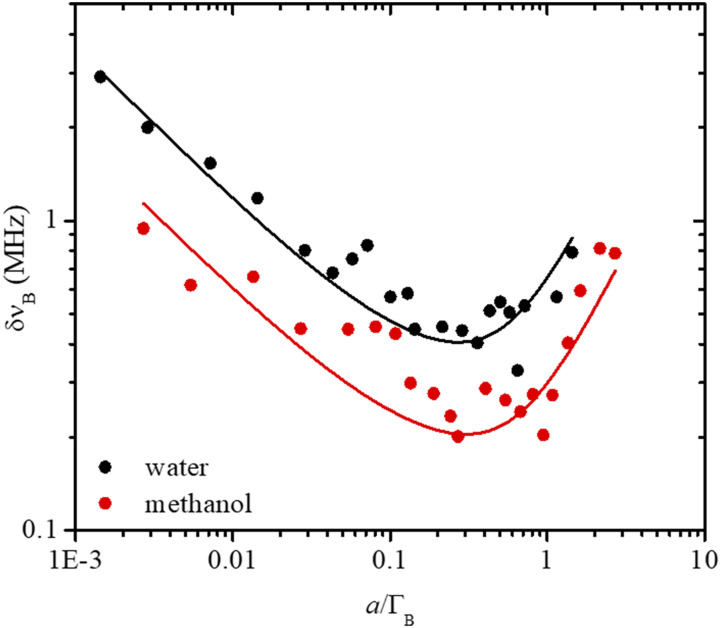
Shift precision δν_B_ evaluated for 100 water (black) and methanol (red) spectra simulated using linewidth, peak amplitude, and noise retrieved from the experiments (Fig. 3), per channel condition. Solid lines represent the fit of the data using Eq. (7).

In Fig. 5, the standard deviation of the peak position is plotted as a function of the normalized channel size *a*/Γ_B_ for water (black) and methanol (red) samples. Data were fitted with the theoretical equation (7), using experimental linewidth Γ_B_ and broadening factor B retrieved from the fit of the linewidth broadening [Fig. 3(a)] with the equation $\Gamma =\sqrt{\Gamma_{B}^{2}+Ba^{2}}$, and considering the same background noise b for both samples. The resulting fitting parameters are Γ_B_ = 270 MHz, N = 8.0 × 10^5^, B = 3.53, and b = 127 for water and Γ_B_ = 144 MHz, N = 8.3 × 10^5^, B = 2.46, and b = 127 for methanol. Data are in good agreement with the localization theory, with precision first monotonically decreasing due to the collection of a higher number of signal photons per channel, and subsequently increasing due to pixelation noise. Since Eq. (7) has been derived considering a Gaussian distribution of photons (see Sec. II B), Gaussian fitting was also tested on the experimental data, leading to comparable results in terms of linewidth broadening and shift precision (data not reported).

In our spectrometer, the maximum precision can be achieved at a channel size about three times smaller than the natural Brillouin linewidth of the sample, which corresponds to about 90 MHz for water and 50 MHz for methanol. In the case of a 5-ms, 2-GHz water(methanol) spectrum acquisition, this corresponds to setting an experiment in which the probe frequency is scanned at 400 GHz/s while 20(40) data points are collected, each of them representing the SBS signal integrated by the LIA for 250(125) *µ*s.

In the case of a system limited to a sampling interval of 2 MHz, as in state-of-the-art SBS spectrometers,^15^ this means that, designing a proper acquisition in which both channel size and sampling interval can be tuned to be 20–50 times larger, precision can be improved of about an order of magnitude while keeping a constant acquisition time. Equivalently, such an improved system will also allow for a 20–50 times faster acquisition at constant SNR and precision.

These results, together with the ones presented in Sec. IV A, experimentally demonstrate the central role, also in SBS, of the spectral channel for maximizing signal collection and optimizing spectra acquisition when considering the spectral characteristics of the sample and the noise properties of the system, as predicted by the localization theory.

## V. CONCLUSIONS

The key feature of localization theory is that the estimation of the location of a sparse signal can be achieved with arbitrary precision given a sufficient number of signal photons and an acquisition system that allows for the maximization of the signal collection.^21^ In Brillouin spectroscopy, extracting the Brillouin shift from the recorded spectra can be understood and treated as a localization problem in the spectral domain, which may take advantage from the localization theory. Interestingly, the localization theory has been naturally applied in spontaneous Brillouin spectroscopy, where the spectrum is collected by a camera^9^ and the spectral PSF is dominated by the etalon dispersion.^2,22^ Instead, stimulated Brillouin scattering (SBS), which has only been recently exploited in biomechanical studies,^15^ is hindered by a suboptimal design of the acquisition parameters and, thus, has shown only modest speed improvements. Applying the localization theory to the SBS spectrum acquisition, we demonstrated the importance of designing a proper detection system to achieve improved performances in terms of precision and speed. We derived the theoretical equation for the precision in the estimation of the Brillouin shift ν_B_ in the case of the frequency-scanning-based SBS spectra acquisition [Eq. (7)], identifying the main noise components, which affect precision and making explicit their dependence on spectral resolution, given by the intrinsic Brillouin linewidth Γ_B_, and channel size *a*, as well as other spectral properties. Through experiments and simulations, we demonstrated that an order of magnitude better precision is expected in water-like samples compared to state-of-the-art SBS spectrometers.^14,15^ Equivalently, a ten times faster acquisition can be achieved without degrading SNR and precision but just collecting ten times less photons with a detection architecture optimized according to the sample under study and the specific noise properties of the system.

This study clearly shows the importance of designing a system in which the channel size can be tuned and matched with the natural linewidth of the sample under study as well as its gain and shift properties. This can be achieved controlling scan speed and LIA time constant in a straightforward manner. However, some remarks should be given regarding the proposed system optimization based on the localization theory. First, the ultimate precision is also affected by other experimental factors such as scan non-linearities and repeatability, frequency, and power stability of the lasers in use. These are technical parameters that need to be optimized in every detection architecture. Second, certain measurements in Brillouin spectroscopy do not fall into the localization model, such as if two closely spaced Brillouin peaks need to be resolved, or if the specific shape of the Brillouin peak is the experimental measurement of interest.^23^

In conclusion, the localization theory has been widely applied in the field of super-resolution microscopy to overcome the diffraction-limited imaging capabilities of the optical systems and obtain precise position determination of individual fluorophores.^20^ Here, we have shown that also in the field of SBS spectroscopy, a proper understanding of the measurement in terms of localization theory can lead to vastly improved performances, thanks to an optimal design of illumination and detection parameters.

## SUPPLEMENTARY MATERIAL

See the supplementary material for supporting content.

## Data Availability

The data that support the findings of this study are available from the corresponding author upon reasonable request.
